# The Brain Proteome of the Ubiquitin Ligase Peli1 Knock-Out Mouse during Experimental Autoimmune Encephalomyelitis

**DOI:** 10.4172/jpb.1000408

**Published:** 2016-09-12

**Authors:** Ragnhild Reehorst Lereim, Eystein Oveland, Yichuan Xiao, Øivind Torkildsen, Stig Wergeland, Kjell-Morten Myhr, Shao-Cong Sun, Frode S Berven

**Affiliations:** 1 Proteomics Unit, Department of Biomedicine, University of Bergen, Norway; 2 Kristian Gerhard Jebsen MS Research Centre, Department of Clinical Medicine, University of Bergen, Bergen Norway; 3 Department of Immunology, The University of Texas MD Anderson Cancer Center, Houston, Texas, USA; 4 Institute of Health Sciences, Shanghai Institutes for Biological Sciences, Chinese Academy of Sciences/Shanghai Jiao Tong University, Shanghai 200031, China; 5 Norwegian Multiple Sclerosis Competence Centre, Department of Neurology, Haukeland University Hospital, Bergen, Norway

**Keywords:** *Peli1* knock-out, Multiple sclerosis, Experimental autoimmune encephalomyelitis (EAE), Brain, TMT-labeling proteomics, Label-free proteomics

## Abstract

The ubiquitin ligase *Peli1* has previously been suggested as a potential treatment target in multiple sclerosis. In the multiple sclerosis disease model, experimental autoimmune encephalomyelitis, *Peli1* knock-out led to less activated microglia and less inflammation in the central nervous system. Despite being important in microglia, *Peli1* expression has also been detected in glial and neuronal cells. In the present study the overall brain proteomes of *Peli1* knock-out mice and wild-type mice were compared prior to experimental autoimmune encephalomyelitis induction, at onset of the disease and at disease peak. Brain samples from the frontal hemisphere, peripheral from the extensive inflammatory foci, were analyzed using TMT-labeling of sample pools, and the discovered proteins were verified in individual mice using label-free proteomics. The greatest proteomic differences between *Peli1* knock-out and wild-type mice were observed at the disease peak. In *Peli1* knock-out a higher degree of antigen presentation, increased activity of adaptive and innate immune cells and alterations to proteins involved in iron metabolism were observed during experimental autoimmune encephalomyelitis. These results unravel global effects to the brain proteome when abrogating *Peli1* expression, underlining the importance of *Peli1* as a regulator of the immune response also peripheral to inflammatory foci during experimental autoimmune encephalomyelitis.

The proteomics data is available in PRIDE with accession PXD003710.

## Introduction

Multiple sclerosis is a chronic inflammatory disease of the central nervous system (CNS) with an estimated prevalence of 2.5 million people worldwide, and it is the most common cause of neurological disability in young adults [1,2]. The cause of the disease is unknown, and research is directed towards finding viable biomarkers for diagnosis monitoring disease activity and therapeutic efficacy [3]. Multiple sclerosis is characterized by inflammatory myelin degradation, and prolonged breakdown leads to permanent demyelination and neuronal cell death, giving rise to a variety of symptoms depending on the affected areas in the CNS [4].

Experimental autoimmune encephalomyelitis (EAE) is a widely used experimental model for inflammatory demyelination in multiple sclerosis research, and when induced by myelin oligodendrocyte glycoprotein peptide (MOG_35-55_) confers both B-cell driven autoantigenic breakdown of myelin and T-cell mediated inflammation [5]. T-cells and antigen presenting cells, such as macrophages and microglia, are reciprocally activated in the CNS, and EAE can be repressed by blocking microglia activation [6]. However, microglia activation may have beneficial effects during CNS injury [7], and a therapeutic challenge is to modulate microglia to promote local recovery [8].

In EAE, the Pellino family E3 polyubiquitin ligase *Peli1* has been shown to be abundantly expressed in microglia, promote microglial activation during the disease course and being necessary for inflammation [9]. *Peli1* expression has also been reported in leukocytes, astrocytes, neurons and oligodendrocyte precursor cells [9] pointing to an essential role for this protein in the CNS. In fact, *Peli1* knockout (KO) mice spontaneously developed autoimmune disease implicating that the *Peli1* protein is a negative regulator of T-cell activation [10]. Intriguingly, *Peli1* KO mice showed an impaired immune cell infiltration [9]. *Peli1* is activated downstream in the Toll-like receptor pathway signalling, and promotes c-IAP mediated degradation of TNF receptor-associated factor 3 (TRAF-3), the latter being an inhibitor of the mitogen-activated protein kinase (MAPK) [11]. In turn, the degradation of TRAF-3 could lead to an increase in the downstream effects following MAPK activation, including activation of the transcription factors NF-κB and AP-1 which induce transcription of inflammatory genes [11]. Furthermore, it has been demonstrated that *Peli1* negatively regulates type 1 interferons in the CNS and that *Peli1* deficiency in mice exposed to VSV infection resulted in reduced brain viral titer and increased survival rate [12].

The inflammation and T-cell infiltration in EAE predominantly occurs in the spinal cord [13], however, pathological changes also occur in brain tissue more remote from the inflammatory lesions [14,15]. By investigating the overall alterations to the brain proteome of *Peli1* KO mice before EAE, at EAE onset and disease peak we sought to reveal additional mechanisms peripheral from the main lesion areas. Thus, the frontal right hemisphere was analyzed using Tandem Mass Tag (TMT) labeling and label-free (LF) proteomics since in this tissue at normal conditions approximately half of the cells are glial and the other half are neurons [16].

## Materials and Methods

Additional information is available in the Supplementary Methods.

### Biological samples

Induction of EAE in *Peli1* KO and WT mice (C57/BL/6 × 129/Sv genetic background) was performed by immunization with MOG_35-55_ as previously described [9]. Animal experiments were conducted in accordance with protocols approved by the Animal Care and Use Committee of the University of Texas MD Anderson Cancer Center. The frontal part of the right cerebral hemisphere was excised, snap-frozen in liquid nitrogen and stored at −80°C until further processing.

### Brain tissue lysis and estimation of protein concentration

Lysis buffer (4% SDS (v/v), 100 mM Tris/HCl pH 7.6, 0.1 M DTT) was added to the brain samples (4 μl/mg) and the samples were homogenized by sonication. The lysates were incubated at 95°C for 3 min, and centrifuged for 5 min at 16000 x g. The protein concentration of the supernatant was estimated by Millipore Direct Detect (Merck Millipore A/S) following the manufacturers protocol.

### Filter aided sample preparation

Proteins from the brain lysates, 50 μg in the TMT-experiment and 20 μg in the label-free (LF) was dissolved in 200 μl 8 M urea in 0.1 M Tris/HCl pH 8.5 for 5 min at room temperature (RT) before it was added to Microcon YM-30 filter units (Millipore, Cat. No. 42410). The brain lysates were digested using filter aided sample preparation (FASP) [17] with the following alterations; the protein to trypsin ratio was 50:1, each washing step was conducted thrice, the sample was added formic acid (FA) to a final concentration of 0.9% prior to Oasis^®^ clean/up.

### Desalting of peptide samples

Samples were acidified to pH 2–3 either by adding FA or by dissolving lyophilized samples directly in 0.1% FA. Reverse phase Oasis^®^ HLB μElution plates 30 μm were used to desalt samples before LC-MS and before TMT labelling. Following lyophilisation, the samples were either dissolved in 5% FA for LC-MS, in Buffer A for Mixed mode fractionation or in 0.1 M TEAB for labelling with TMT 10-plex reagents.

### TMT labeling of samples

One TMT 10-plex label reagent set (Thermo Scientific, product no: 90110B) was divided in two 10-plex experiments that were to be combined in the data analysis. The brain lysate samples (n = 38) from the six conditions WT 0, WT 10, WT 20, KO 0, KO 10 and KO 20 were pooled so that each condition was represented by three small pools consisting of 1–3 individuals in each. The numbers 0, 10 and 20 in the names of the conditions indicate the number of days post infection (dpi) that the mice were euthanized and the brain were sampled. Two identical reference pools off all 18 pools were generated giving 20 samples (Supplementary Methods). The samples were tagged by a TMT-10 plex split in two, desalted, fractionated by mixed mode HPLC and analyzed using an Orbitrap Elite (Thermo Scientific) mass spectrometer (Supplementary Methods).

### TMT data quantification in proteome discoverer

LC-MS data from the two TMT-10 plex experiments were analyzed in Proteome Discoverer 1.4 (Thermo Scientific) using Sequest and MS Amanda (version 1.4.4.2822).

All samples were divided by the reference sample within each TMT 10-plex using Proteome Discoverer. Only unique peptides were used for quantification, and no co-isolation threshold was set. The results were filtered such to include only proteins identified with high confidence peptides (FDR <1%) and 5–20 amino acids prior to export. In Microsoft Excel the protein abundances were log_2_ transformed and each condition was normalized within the condition by subtracting the respective median value before further analyses. Following normalization, the two TMT datasets were combined using the vlookup function. The TMT labeling strategy of small pools resulted in quantification of 5264 and 5261 proteins (≥ 3 peptide spectrum matches, PSMs) in TMT 10-plex experiment one and two, respectively. Of these, 4874 proteins were quantified in both experiments. A two tailed t-test was used for pairwise comparisons of the protein levels between conditions and a Benjamini-Hochberg p-value correction was applied to correct for multiple comparisons, no p-values were significant (q<0.05). Resultantly, proteins with a significant t-test (p-value ≤0.05) and with an average fold change (FC) ≥ 20% (log_2_ FC ≥ 0.263, ≤ −0.263), approximately 2 standard deviations (SD) from the mean of the calculated ratios, were considered significantly different. As the sample distribution approximates a normal distribution, the 2 SD cutoff means that 95.45% of the fold changes in the dataset were excluded by this filter.

### Label-free analysis

Brain lysates from the 38 individual mice and four reference samples (pool of equal protein amount from all the 38 individual lysates) were digested by FASP, desalted using Oasis^®^ plates and prepared for LF analysis using an Orbitrap Elite and Progenesis QI v2.0 (Nonlinear Dynamics). We applied 12 different LC-MS methods and duplicate injections to the reference samples in order to increase the identification rate of low-abundant peptides (Supplementary Methods).

The protein intensities for each of the individual samples were divided by the median intensity of the reference samples, and these ratios were log_2_ transformed prior to statistical analyses and FC calculations. A two-tailed t-test was performed as described for the TMT experiment, no proteins were significantly different (q<0.05) after Benjamini-Hochberg correction of multiple comparisons, and the same p-value cutoff filter was applied on the t-test as in the TMT experiment. The proteins had to be quantified with more than 1 peptide to be reported. Proteins identified in protein groups were excluded, unless otherwise stated. The FC values in the label free experiment were generally larger than those observed in the TMT experiment, most likely due to the ion ratio compression commonly observed in TMT and iTRAQ experiments [18]. The FC cut off was set to ≥40% (log_2_ FC ≥ 0.485, ≤ −0.485), approximately 2 SD from the mean of the calculated ratios.

### Publication of proteomics data in PRIDE

The proteomics raw files and search/quantification results have been deposited to the ProteomeXchange Consortium [19] via the PRIDE partner repository [20] with the dataset identifier PXD003710.

## Results and Discussion

### TMT and label-free quantification

Small pools (n=18 pools) comprised of a total of 38 samples containing protein extracts from the frontal right hemisphere of *Peli1* KO and WT mice harvested before EAE induction (0), at inflammation onset (10 dpi), and at disease peak (20 dpi) were TMT-labeled and analyzed by mass spectrometry. The analysis of the generated data resulted in quantification of 4874 proteins, of which 187 were significantly regulated between one (or) more of the six conditions compared.

**Figure 1:**
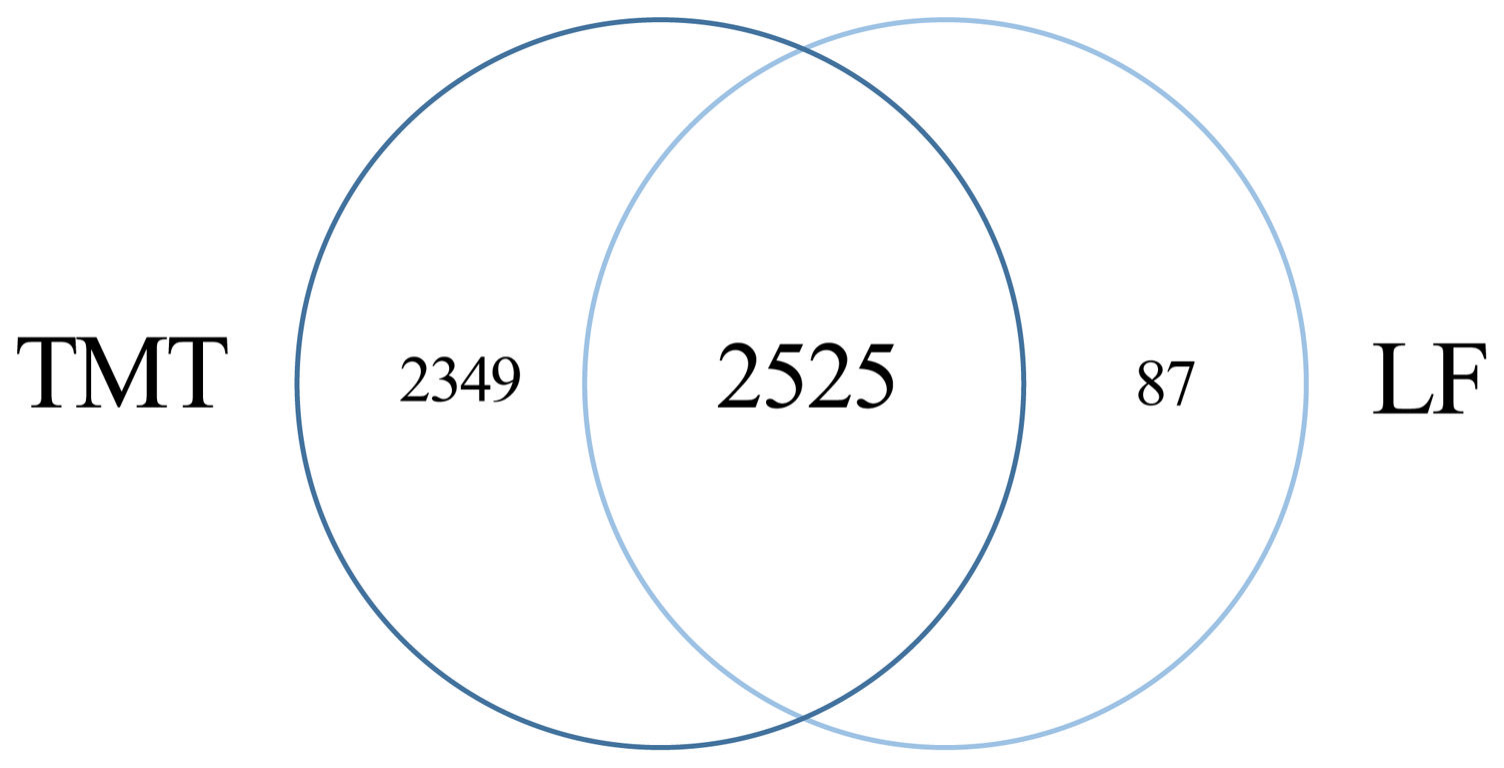
Proteins quantified in brain samples from *Peli1* KO and WT mice The number of proteins quantified in both experiments with ≥3 PSMs in both of the TMT experiments and >1 unique peptide in the LF experiment. Proteins identified in protein groups were excluded.

A LF experiment was conducted to verify in individual mice the regulation observed from the pools used in TMT-experiment. The LF strategy on all individual samples (n=38) used as pools in the TMT experiment above resulted in quantification of 2612 proteins, and 2525 of these were also quantified in the LF experiment (Figure 1). The Pearson correlation of the fold change for the 2525 proteins between KO and WT in TMT vs. LF were 0.52 for timepoint 0, 0.62 for 10 dpi, and 0.76 for 20 dpi. This correlation is as expected considering the different nature of the two methods where label free tend to give a broader distribution of ratios compared to TMT due to compression effects when using TMT [18].

**Figure 2:**
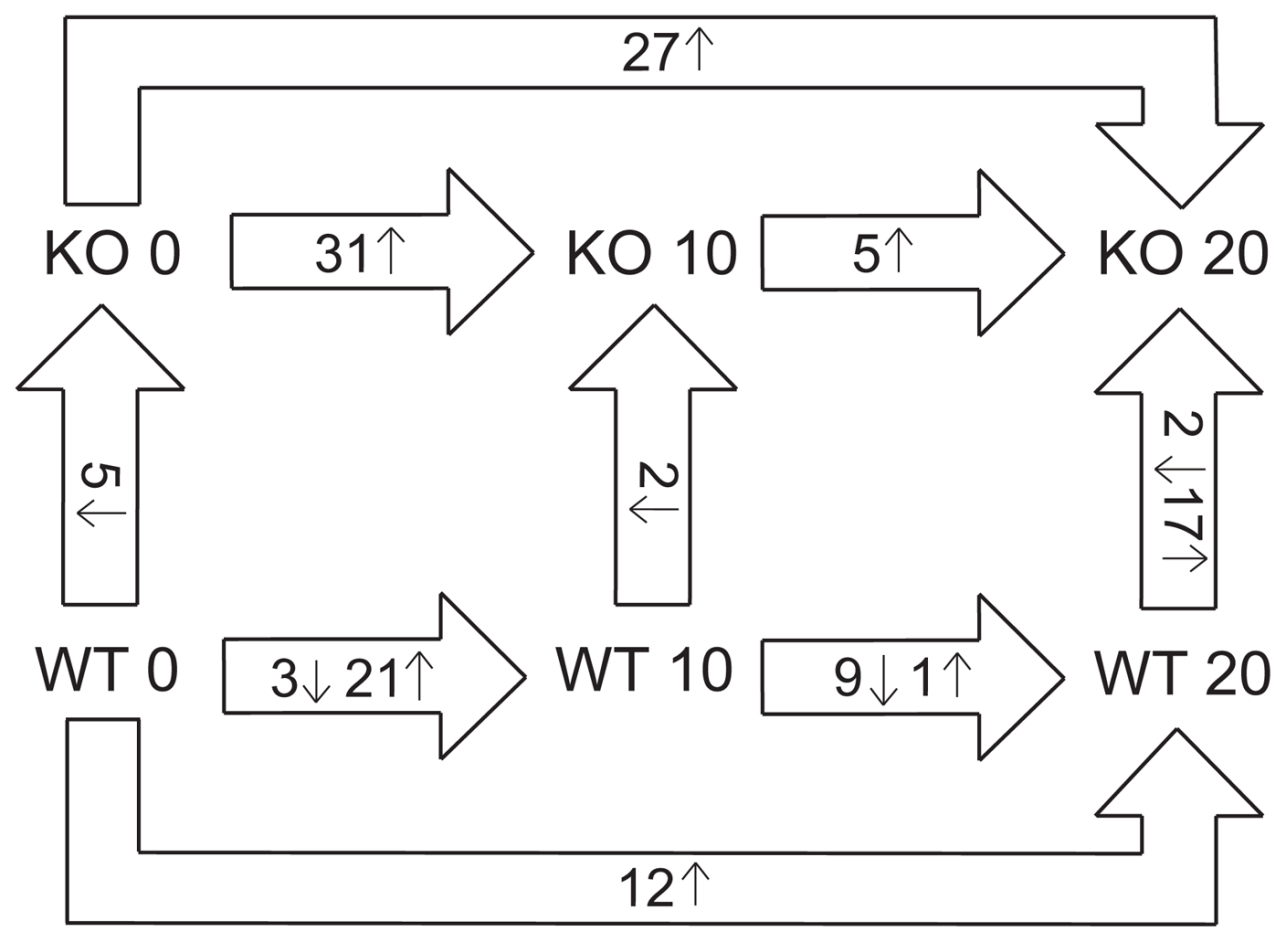
Comparisons of regulated proteins between *Peli1* KO and WT and between timepoints The number of proteins significantly regulated between conditions (p ≤ 0.05, TMT log_2_ Fold change (FC) ≥ 0.263, ≤ −0.263, LF log_2_ FC ≥ 0.485, ≤ −0.485) and their regulation are indicated by arrows. Horizontal arrows: proteins significantly regulated between the given timepoints in KO or in WT, respectively. Large vertical arrows: number of proteins significantly regulated between KO and WT at the given timepoints. Arrowheads indicate the nominator in FC calculations. The figure should be read as follows; between KO 0 and KO 10 a total of 31 proteins were significantly different and all were more abundant in KO 10 as indicated by the arrow pointing upwards. KO 0: *Peli1* knock out prior to EAE immunization, KO 10: *Peli1* knock out 10 days after EAE immunization, KO 20: *Peli1* knock out 20 days after EAE immunization, WT 0: Wild-type before EAE immunization, WT 10: Wild-type 10 days after EAE immunization, WT 20: Wild type 20 days after EAE immunization.

Of the total of 187 proteins found regulated from the TMT-experiment, 80 proteins were also quantified in the LF experiment, and 57 of these were also significantly regulated between one or more conditions in the LF experiment. These proteins formed the main basis for further interpretation of the proteomics results. Most differences in protein regulation between KO and WT were found at 20 dpi (Figure 2) when the difference in EAE clinical scores was highest (Supplementary Figure 1). Thus 20 dpi was the main focus in the analyses in the following sections.

The data analyses of all quantified proteins in TMT and LF experiments are available in Supplementary Table 1. All the significant protein regulations in both TMT and LF experiments between *Peli1* KO and WT are presented in Tables 1–3. Furthermore, all the significant protein regulations in both TMT and LF experiments between the different timepoints (0, 10 dpi and 20 dpi) in *Peli1* KO and between the different timepoints in WT, respectively, are listed in Supplementary Tables 2–4.

### Complement components were more abundant in *Peli1* KO compared to WT at 20 dpi

**Figure 3:**
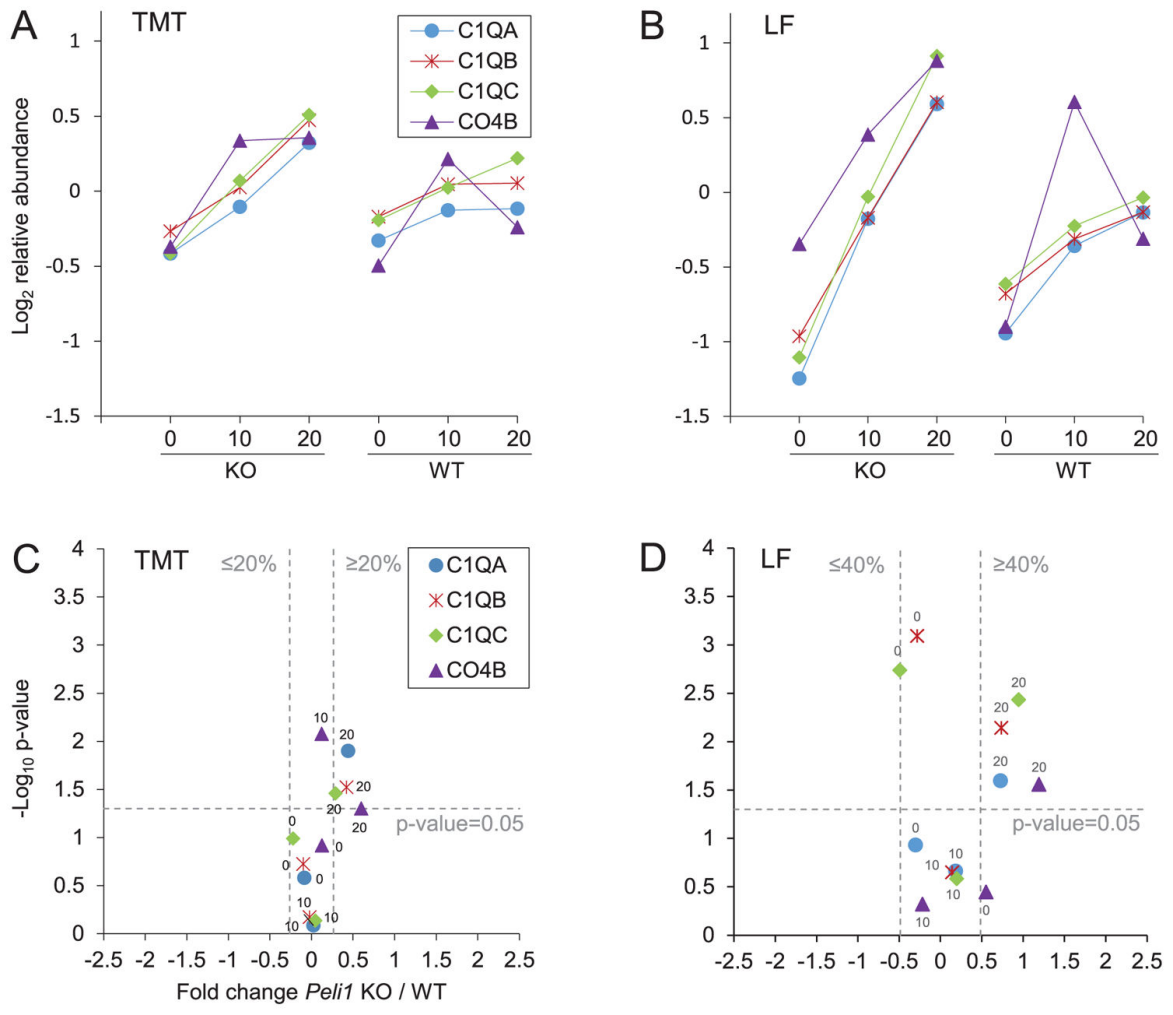
Complement proteins C1QA-C and CO4b were more abundant in *Peli1* KO 20 compared to WT 20 The proteins are represented by their protein short names; complement C1q subcomponent A-C (C1QA-C), complement C-4B (CO4B). The legend and y-axis title in figure A is also valid for B, and in C is also valid for D. A) The figure shows the average protein abundance in the TMT experiment for the indicated protein after 0, 10 and 20 dpi for KO and WT, respectively. N=3 pools each condition. B) The figure shows the average protein abundance in the LF experiment after 0, 10 and 20 dpi for KO and WT, respectively. N=5–7 individual animals each condition. C) The indicated protein the average TMT fold change (FC) value and the t-test p-value at timepoint 0 (KO 0/WT 0) have been plotted in a volcano plot and indicated with “0”, at timepoint 10 dpi (KO 10/WT 10) with “10” and at timepoint 20 dpi (KO 20/WT 20) with “20”. Stapled grey lines indicate the significance thresholds for the TMT experiment (p ≤ 0.05, log_2_ FC KO/WT ≥ 0.263, ≤ −0.263), such that significant regulations will distribute in the upper left area (downregulated) and upper right area (upregulated) in the plot. D) Same as in C but for the LF experiment, and the stapled grey lines indicate the significance thresholds for the LF experiment (p ≤ 0.05, log_2_ FC ≥ 0.485, ≤ −0.485). Note that C1QB was quantified as the leading protein of a protein group in the LF experiment. The average abundances and SD for each of the regulated proteins are available in Supplementary Table 5.

The complement subcomponents C1q A-C and the complement component C4b (CO4B) were significantly higher in KO 20 compared to WT 20 in the TMT experiment and verified in the LF (Figure 3). Notably, complement components C1q A-C are parts of the C1 complex that binds antibodies and have been shown to contribute to synaptic pruning in the CNS [21], and are expressed by several cells in the CNS including microglia [22]. Other downstream components in the complement cascade were downregulated during EAE from 10 dpi to 20 dpi in both KO and WT (data available in Supplementary Table 1), indicating a cascade-independent role for these subunits.

### Monocyte surface markers and astrocyte associated proteins were more abundant in *Peli1* KO than WT at 20 dpi

**Figure 4:**
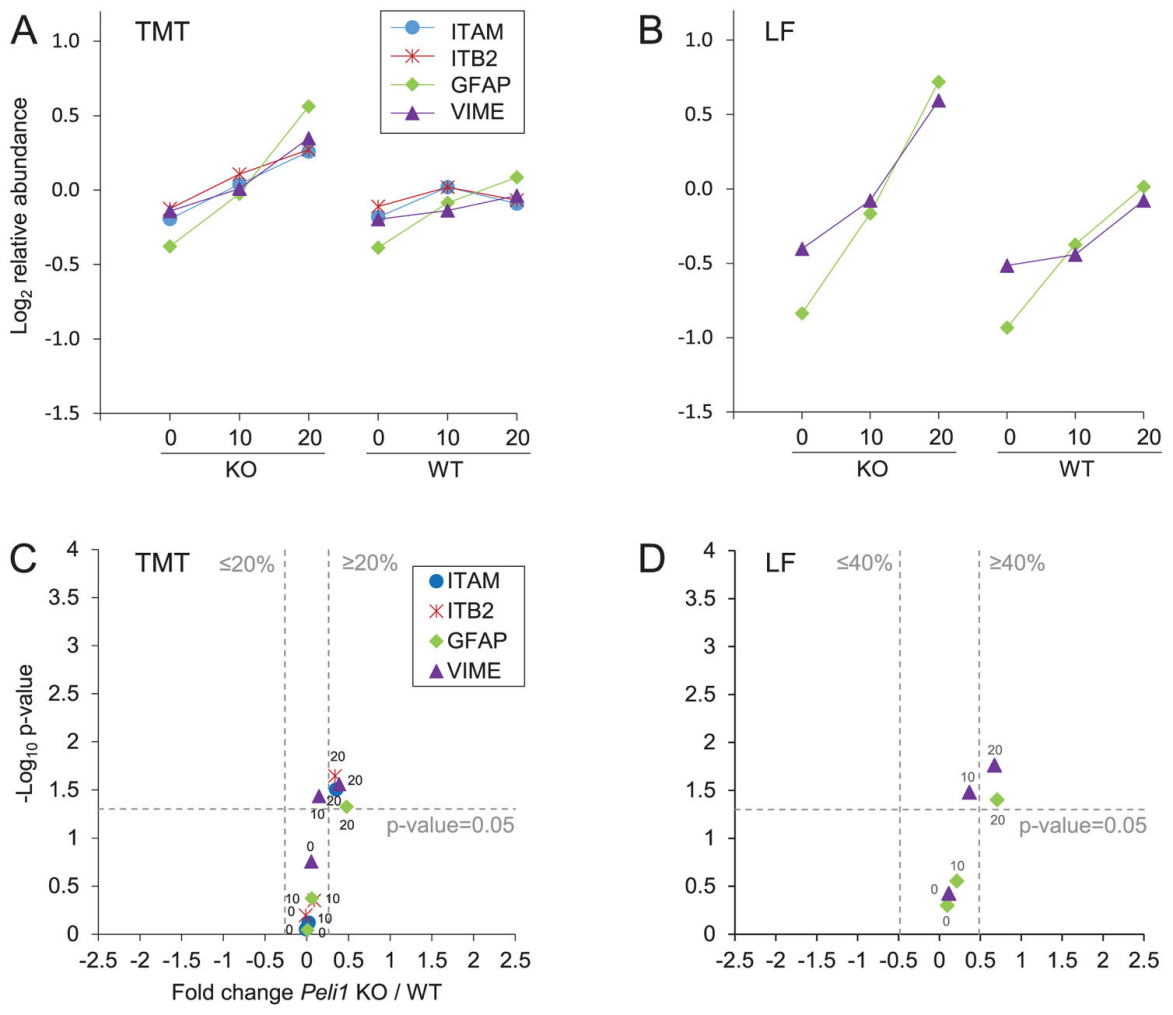
The integrins ITAM and ITB2, and astrocyte associated proteins GFAP and VIME were more abundant in *Peli1* KO 20 compared to WT 20 The proteins are represented by their protein short names; integrin alpha-M (ITAM), integrin beta-2 (ITB2), glial fibrillary acidic protein (GFAP), vimentin (VIME). See Figure 3 for a detailed figure legend. The figure shows the average protein abundance in KO and WT, respectively, in the TMT experiment (A) and in the LF experiment (B). Volcano plots of KO/WT protein ratios at different timepoints in the TMT experiment (C) and in the LF experiment (D) are shown. Note that ITAM and ITB2 were not detected in the LF experiment. The average abundances and SD for each of the regulated proteins are available in Supplementary Table 5.

Integrin alpha-M (ITAM, CD11b), a commonly used lymphocyte and monocyte surface marker, was significantly more abundant in KO 20 than WT 20 in the TMT experiment (Figure 4). ITB2 (CD18) was upregulated in KO 20 and is a common binding partner to ITAM, and together they form the complement receptor 3 (CR3), that is expressed on lymphocytes and monocytes [23]. The protein plastin-2 (PLP2) had significantly higher levels in KO 20, and was confirmed by the LF analysis. PLP2 is involved in the activation of T-cells and localization of T-cell surface markers, but is also expressed in monocytes and B-lymphocytes and certain cancers [24]. Thus, these proteins indicated an increased number- or activity of immune cells in *Peli1* KO 20 compared to WT 20. LYN, a negative regulator of B-cells and myeloid cells, was also more abundant in KO 20 compared to WT 20. Notably, loss of function of this protein in B-cells induces autoimmunity [25], and upregulation of this protein might serve to limit B-cell activity in the brains of *Peli1* KO mice at 20 dpi.

The intermediate filaments GFAP and VIME increased in both KO and WT during EAE progression, but were significantly more abundant in KO 20 (Figure 4), indicating increased astrocyte activity in KO. Previous studies have shown that GFAP is associated with disease severity in EAE [26], and that the protein increased in abundance following relapses in the CSF of multiple sclerosis patients [27]. Notably, astrocytes have both protective and pro-inflammatory effects in the CNS [28]. As *Peli1* KO mice had a decreased clinical score compared to WT at 20 dpi, the increase of GFAP might be mirroring protective effects of astrocytes in the CNS of the *Peli1* KO mice.

### Interferon signaling related proteins were more abundant in *Peli1* KO than WT at 20 dpi

Several proteins related to interferon signaling were among the most upregulated in *Peli1* KO 20 compared to WT 20, such as IFIT3, IRGM1 and the GTPases IIGP1, GBP2 and GBP4 (Table 3). These proteins were among the most regulated proteins and have been demonstrated to be upregulated by interferon signaling [29–34]. Previous studies have shown increased abundance of transcription factor STAT1, known to induce expression of interferons, in spinal cords of EAE mice [35], as observed in both KO and WT brains, albeit most abundant in KO 20 compared to WT 20.

### Proteins involved in antigen presentation were more abundant in *Peli1* KO than WT at 20 dpi

**Figure 5:**
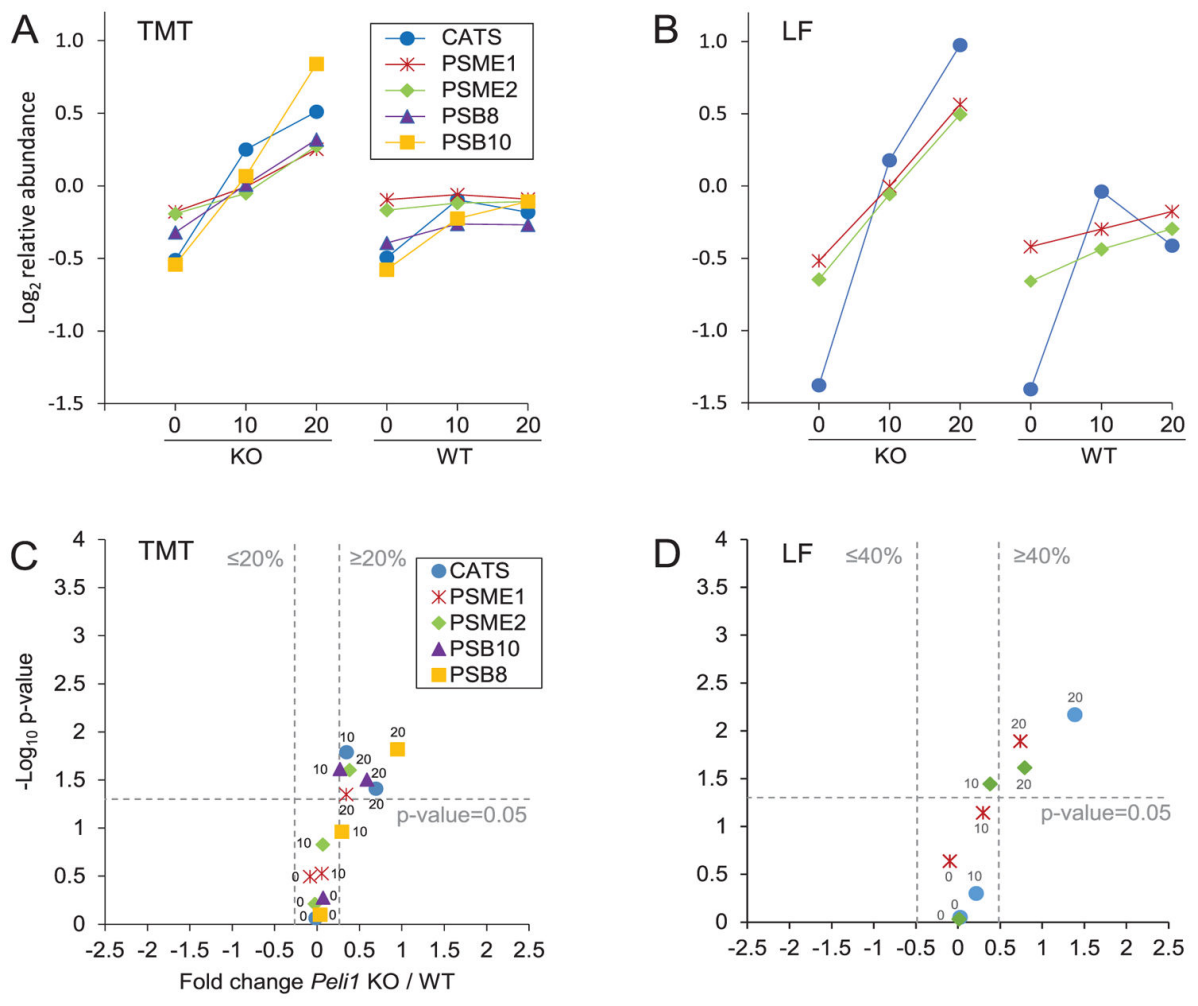
Proteins linked to antigen presentation were more abundant in *Peli1* KO 20 than WT 20 The proteins are represented by their protein short names; cathepsin S (CATS), proteasome activator complex subunit 1 (PSME1), proteasome activator complex subunit 2 (PSME2), proteasome subunit beta-10 (PSB10), proteasome subunit beta-8 (PSB8). See Figure 3 for a detailed figure legend. The figure shows the average protein abundance in KO and WT, respectively, in the TMT experiment (A) and in the LF experiment (B). Volcano plots of KO/WT protein ratios at different timepoints in the TMT experiment (C) and in the LF experiment (D) are shown. Note that PSB8 and PSB10 were not detected in the LF experiment. The average abundances and SD for each of the regulated proteins are available in Supplementary Table 5.

**Figure 6:**
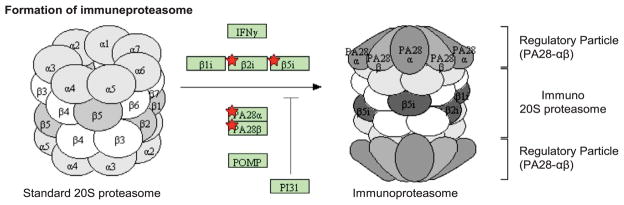
Proteins upregulated in *Peli1* KO 20 in immunoproteasome formation An overlay of the proteins significantly different between KO 20 and WT 20 in the TMT experiment mapped to the ≪proteasome≫ pathway in KEGG (DAVID, Benjamini Hochberg q-value=0.016). Gene products from the TMT dataset are shown with red stars. PA28a (PSME1), PA28b (PSME2), b2i (PSMB10) and b5i (PSMB8).

CATS, a protein known to remove the invariant chain from MHC class II molecules prior to antigen loading, was significantly more abundant in KO 20 than WT 20. Similarly, proteins PSME1, PSME2, PSB8 and PSB10 were significantly more abundant in KO 20. The upregulation of CATS as well as PSME1 and PSME2 was confirmed in the LF experiment (Figure 5). Proteasome subunits PSB8 and PSB10 and proteasome activator complex subunits PSME1 and PSME2 were, in the TMT experiment, all significantly more abundant in KO 20. These proteins are subunits of the immunoproteasome or proteins that facilitate its formation (Figure 6) [36]. Altogether, this indicated a higher degree of antigen presentation in *Peli1* KO at 20 dpi.

### Proteins significantly different prior to EAE induction indicate differences in iron availability between *Peli1* KO and WT

**Figure 7:**
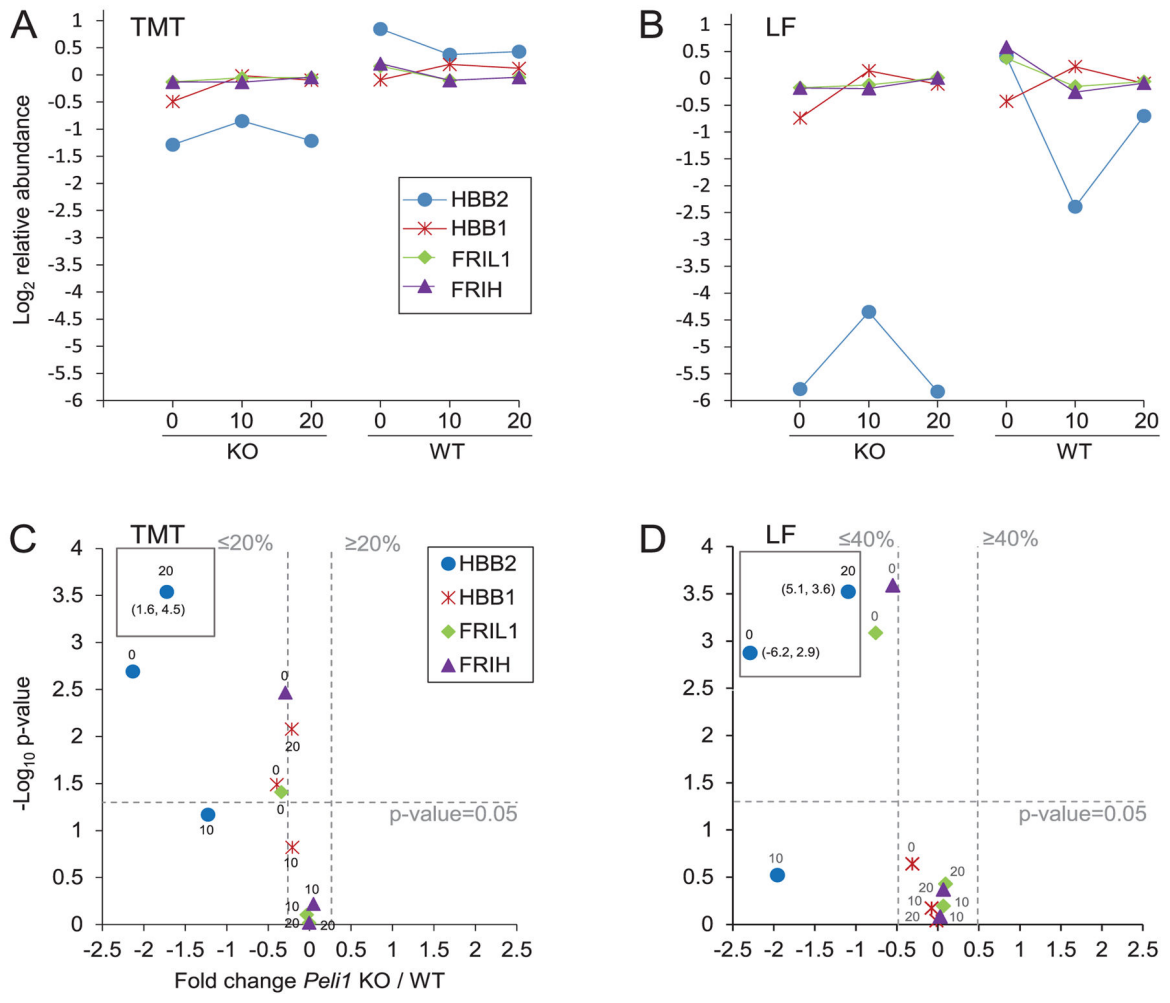
Iron associated proteins were less abundant in *Peli1* KO 0 than WT 0 Proteins are represented by their short names; Hemoglobin subunit beta-2 (HBB2), Hemoglobin subunit beta-1 (HBB1), Ferritin light chain (FRIL1), Ferritin heavy chain (FRIH). See Figure 3 for a detailed figure legend. The figure shows the average protein abundance in KO and WT, respectively, in the TMT experiment (A) and in the LF experiment (B). Volcano plots of KO/WT protein ratios at different timepoints in the TMT experiment (C) and in the LF experiment (D) are shown. The average abundances and SD for each of the regulated proteins are available in Supplementary Table 5.

The hemoglobin subunits HBB2, HBB1 were significantly less abundant in *Peli1* KO prior to EAE induction. These proteins bind iron through their heme prosthetic group. Similarly, FRIH and FRIL1 ferritin proteins important for storing iron, were also significantly lower in KO 0 (Figure 7). Previous studies have reported that iron storage may be affected by inflammation [37], and ferritin has been reported upregulated in the CSF of patients with active multiple sclerosis [38]. TFR1, a receptor for transferrin that transfers iron to iron stores in the body, was more abundant in KO before EAE induction (significant in LF) and higher throughout EAE. These differences in iron related proteins may also influence the synthesis of myelin [39], and we observed that the myelin protein PLP1 had significantly lower abundance (p ≤ 0.05) in KO than WT at all-time points in both TMT and LF experiments albeit with a moderate FC (LF FC ≤ −0.13, TMT FC ≤ −0.20).

The hemoglobin subunit HBB2 was the most significantly regulated protein at 20 dpi and less abundant in KO in both TMT and LF. Notably, other hemoglobin subunits were not significantly different between KO and WT 20 dpi (data available in Supplementary Table 1). Previous studies have shown certain hemoglobin subunits to be expressed by certain neurons and glia affecting mitochondrial function [40], and the human hemoglobin beta was upregulated in mitochondrial fractions of multiple sclerosis post mortem brains [41]. Thus the observed differences in HBB2 between KO and WT might involve other cell types and mechanisms than oxygen transfer in blood.

### Indications of increased activity of B-cells in *Peli1* KO

**Figure 8:**
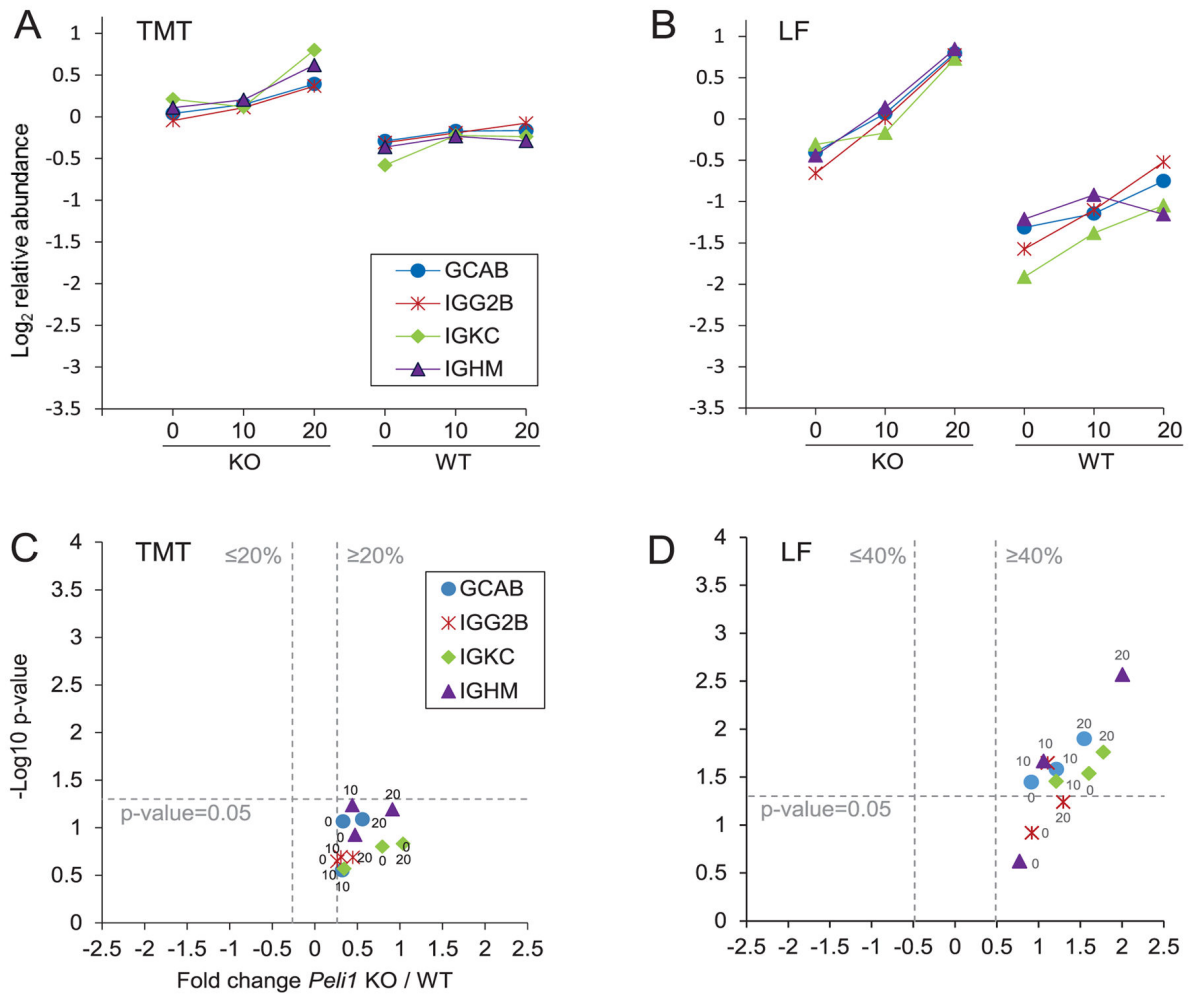
Immunoglobulins were more abundant in LF when comparing *Peli1* KO 20 dpi to WT 20 dpi The proteins are represented by their protein short names; Ig gamma-2A chain C region secreted form (GCAB), Ig gamma-2B chain C region (IGG2B), Ig kappa chain C region (IGKC), Ig mu chain C region (IGHM). See Figure 3 for a detailed figure legend. The figure shows the average protein abundance in KO and WT, respectively, in the TMT experiment (A) and in the LF experiment (B). Volcano plots of KO/WT protein ratios at different timepoints in the TMT experiment (C) and in the LF experiment (D) are shown. The average abundances and SD for each of the regulated proteins are available in Supplementary Table 5.

The immunoglobulin proteins GCAB and IGKC were significantly higher in *Peli1* KO and WT mice at all timepoints (0, 10 dpi and 20 dpi) in LF, verifying a trend seen in the TMT experiment. Furthermore, IGHG3 and IGHM were significantly more abundant in KO 20 compared to WT 20 in the LF experiment and the same trend in TMT (Figure 8). Together, the expression pattern of these immunoglobulin constant regions indicated an increased activity of B-cells in the brains of *Peli1* KO mice.

### Roles of *Peli1* in immunity and inflammation

The role of *Peli1* in immunity is paradoxical, where genetic knock-out leads to a higher degree of peripheral autoimmunity [10], but to less secretion of inflammatory cytokines and beneficial clinical scores in the autoimmune encephalomyelitis model EAE [9]. The knock-out of *Peli1* has also been shown to cause an increased production of type-1 interferons α and β in the brain in response to infection, leading to increased immunity and survival [12]. In EAE endogenous Type I interferons are considered to be anti-inflammatory and neuroprotective [42], and interferon- β is successfully administered to multiple sclerosis patients to prevent relapses in relapsing-remitting disease [43]. The type 1 interferons have been shown to increase the degree of antigen presentation and initiate B- and T-cell responses including antibody secretion [44]. This is in line with our observation that upregulated proteins in *Peli1* deficient mice at 20 dpi potentially favored antigen presentation (PSME1, PSME2, CATS) and increased activity of different immune cells (ITAM, ITB2, PLP2, immunoglobulins). As discussed above, several proteins downstream of both type I and type II interferon signaling pathways (IFIT3, ITGM1 and GTPases) were upregulated in *Peli1*-KO at 20 dpi.

We discovered that relatively few proteins were regulated in the frontal part of the brain as a result of the Peli-1 deficiency. The highest number of regulations was observed at EAE disease peak at (20 dpi) and could be associated with an activated immune response as discussed above. As EAE is an autoimmune disease, one would hypothesize that a further increased immune response would be damaging to the host and associated with a worse clinical outcome. However, it seems like these processes might be beneficial as decreased EAE clinical scores are observed for *Peli1* deficient mice. The EAE clinical scores were based on disability of movement largely reflecting damages in the spinal cord and other disabilities not monitored (cognitive, visual/auditory) might be affected in the *Peli1* deficient mice.

In microglia *Peli1* promotes degradation of TRAF-3 (inhibitor of MAPK) resulting in MAPK activation of AP1 and transcription of inflammatory genes [11]. Several proteins downstream of *Peli1* were detected in the TMT and/or LF datasets, namely TRAF3 and the MAPK family members JNK (MK8-10), ERK (MK3, MK1) and p38 (MK11, MK14) but they had not significantly different levels in *Peli1*-KO (data available in Supplementary Table 1). The reason that TRAF3 was not downregulated and MAPK not upregulated in the *Peli1*-KO in our datasets could be due to averaging effects from analyzing the sum of all cell types in the entire frontal right hemisphere. Of note, some of the protein abundances in our study could have been affected by an increased peripheral inflammation from the meninges covering the brain. Peripheral influences on protein abundances caused by blood contamination were unlikely as most hemoglobin subunits were unregulated as discussed above.

## Conclusion

In conclusion, we have revealed the overall proteomic effect of *Peli1* KO on the frontal right hemisphere during EAE and discovered several upregulated proteins potentially favoring antigen presentation (PSME1, PSME2, CATS) and increased activity of different immune cells (ITAM, ITB2, PLP2, immunoglobulins) and astrocytes (GFAP). Whether these regulated proteins are directly involved in protecting the mice from EAE or reflect less advantageous consequences is, however, not clear. Further studies of *Peli1* interaction partners and cell-type specific signaling pathways are needed to specifically modulate the effects observed by *Peli1* KO to introduce new therapies to treat neurodegenerative diseases like multiple sclerosis.

## Supplementary Material

Supp Table 1

Supp information

supp fig

supp methods

supp table 2

supp table 3

supp table 4

supp table 5

## Figures and Tables

**Table 1:** Proteins regulated in *Peli1* KO 0 compared to WT 0.

			TMT		LF	
Accession	Description	Name	p-value	FC	p-value	FC
P02089	Hemoglobin subunit beta-2	HBB2	0.002	−2.13	0.001	−6.19
Q8BMF3	NADP-dependent malic enzyme, mitochondrial	MAON	0.04	−0.69	0.03	−1.34
P29391	Ferritin light chain 1	FRIL1	0.04	−0.34	0.0008	−0.76
P35505	Fumarylacetoacetase	FAAA	0.04	−0.32	0.01	−0.74
P09528	Ferritin heavy chain	FRIH	0.003	−0.29	0.0003	−0.55
Q9EQG7	Ectonucleotide pyrophosphatase/ phosphodiesterase family member 5	ENPP5	0.02	−0.50	ND	ND
Q9CWN7	CCR4-NOT transcription complex subunit 11	CNO11	0.04	−0.36	ND	ND
O09159	Lysosomal alpha-mannosidase	MA2B1	0.004	−0.34	ND	ND
Q9JMD3	PCTP-like protein	PCTL	0.01	−0.31	ND	ND
P54869	Hydroxymethylglutaryl-CoA synthase, mitochondrial	HMCS2	0.03	−0.30	ND	ND
A3KMP2	Tetratricopeptide repeat protein 38	TTC38	0.01	−0.29	ND	ND
Q6A078	Centrosomal protein of 290 kDa	CE290	0.02	−0.28	ND	ND
Q9D7V9	N-acylethanolamine-hydrolyzing acid amidase	NAAA	0.003	0.28	ND	ND
D3Z7H4	Germ cell-specific gene 1-like protein	GSG1L	0.01	0.28	ND	ND
O88829	Lactosylceramide alpha-2,3-sialyltransferase	SIAT9	0.03	0.28	ND	ND
P55065	Phospholipid transfer protein	PLTP	0.04	0.29	ND	ND
O70496	H(+)/Cl(−) exchange transporter 7	CLCN7	0.03	0.32	ND	ND
P50172	Corticosteroid 11-beta-dehydrogenase isozyme 1	DHI1	0.02	0.44	ND	ND
Q9DCC7	Isochorismatase domain-containing protein 2B, mitochondrial	ISC2B	0.02	0.73	ND	ND

Proteins significantly different (p-value ≤ 0.05; TMT FC ≥ 20% (log_2_ FC ≥ 0.263, ≤ −0.263); LF FC ≥ 40% (log_2_ FC ≥ 0.485, ≤ −0.485)) between KO and WT before EAE induction (0) sorted by FC. ND, Not detected in the LF experiment.

**Table 2:** Proteins regulated in *Peli1* KO 10 compared to WT 10.

			TMT		LF	
Accession	Description	Name	p-value	FC	p-value	FC
Q8BG18	N-terminal EF-hand calcium-binding protein 1	NECA1	0.01	−0.35	0.02	−0.74
Q61646	Haptoglobin	HPT	0.02	−0.34	0.01	−0.67
Q9EQG7	Ectonucleotide pyrophosphatase/ phosphodiesterase family member 5	ENPP5	0.01	−0.35	ND	ND
Q8CFE6	Sodium-coupled neutral amino acid transporter 2	S38A2	0.001	−0.33	ND	ND
O35955	Proteasome subunit beta type-10	PSB10	0.02	0.27	ND	ND
Q8BSY0	Aspartyl/asparaginyl beta-hydroxylase	ASPH	0.05	0.31	ND	ND
Q6ZWQ0	Nesprin-2	SYNE2	0.05	0.32	ND	ND

Proteins significantly different (p-value ≤ 0.05; TMT FC ≥ 20% (log_2_ FC ≥ 0.263, ≤ −0.263); LF FC ≥ 40% (log_2_ FC ≥ 0.485, ≤ −0.485)) between KO 10 and WT 10 sorted by FC. ND: Not detected in the LF experiment.

**Table 3:** Proteins regulated in *Peli1* KO 20 compared to WT 20.

			TMT		LF	
Accession	Description	Name	p-value	FC	p-value	FC
P02089	Hemoglobin subunit beta-2	HBB2	0.00004	−1.64	0.0003	−5.13
Q00897	Alpha-1-antitrypsin 1-4	A1AT4	0.03	−0.81	0.004	−2.02
Q8BGB7	Enolase-phosphatase E1	ENOPH	0.01	0.31	0.0004	0.54
P26041	Moesin	MOES	0.01	0.29	0.006	0.60
P20152	Vimentin	VIME	0.03	0.39	0.017	0.67
P03995	Glial fibrillary acidic protein	GFAP	0.05	0.48	0.04	0.71
P98086	Complement C1q subcomponent subunit A	C1QA	0.01	0.44	0.025	0.73
P14106	Complement C1q subcomponent subunit B*	C1QB	0.03	0.42	0.0007	0.74
P97371	Proteasome activator complex subunit 1	PSME1	0.04	0.34	0.013	0.74
P97372	Proteasome activator complex subunit 2	PSME2	0.02	0.38	0.024	0.79
Q61233	Plastin-2	PLSL	0.02	0.49	0.013	0.85
P37804	Transgelin	TAGL	0.02	0.51	0.024	0.86
Q02105	Complement C1q subcomponent subunit C	C1QC	0.03	0.29	0.004	0.95
P01029	Complement C4-B	CO4B	0.05	0.60	0.028	1.19
O70370	Cathepsin S	CATS	0.04	0.69	0.007	1.39
Q60766	Immunity-related GTPase family M protein 1	IRGM1	0.03	1.19	0.012	1.73
P42225	Signal transducer and activator of transcription 1	STAT1	0.04	0.86	0.005	2.00
Q9Z0E6	Interferon-induced guanylate-binding protein 2	GBP2	0.04	0.95	0.014	2.09
Q9QZ85	Interferon-inducible GTPase 1	IIGP1	0.03	1.89	0.002	3.21
Q8K207	Uncharacterized protein C1orf21 homolog	CA021	0.02	−0.69	ND	ND
Q8R1B5	Complexin-3	CPLX3	0.03	−0.36	ND	ND
Q5F2E7	Nuclear fragile X mental retardation-interacting protein 2	NUFP2	0.01	−0.33	ND	ND
Q9CXJ1	Probable glutamate--tRNA ligase, mitochondrial	SYEM	0.03	−0.29	ND	ND
Q5XJY4	Presenilins-associated rhomboid-like protein, mitochondrial	PARL	0.02	−0.27	ND	ND
P48455	Serine/threonine-protein phosphatase 2B catalytic subunit gamma isoform	PP2BC	0.03	0.26	ND	ND
P25911	Tyrosine-protein kinase Lyn	LYN	0.03	0.33	ND	ND
P11835	Integrin beta-2	ITB2	0.02	0.34	ND	ND
P05555	Integrin alpha-M	ITAM	0.03	0.35	ND	ND
Q05144	Ras-related C3 botulinum toxin substrate 2	RAC2	0.04	0.51	ND	ND
O35955	Proteasome subunit beta type-10	PSB10	0.03	0.59	ND	ND
Q61107	Guanylate-binding protein 4	GBP4	0.04	0.78	ND	ND
P28063	Proteasome subunit beta type-8	PSB8	0.02	0.95	ND	ND
Q64345	Interferon-induced protein with tetratricopeptide repeats 3	IFIT3	0.04	1.31	ND	ND

Proteins significantly different (p-value ≤ 0.05; TMT FC ≥ 20% (log_2_ FC ≥ 0.263, ≤ −0.263); LF FC ≥ 40% (Log_2_ FC ≥ 0.485, ≤ −0.485)) between KO 20 and WT 20 sorted by FC. ND: Not detected in the LF experiment.

* LF quantification of protein group.

## Abbreviations

EAE: Experimental Autoimmune Encephalomyelitis
KO: Knock Out
WT: Wild-Type
CNS: Central Nervous System
APC: Antigen Presenting Cells
FASP: Filter Aided Sample Preparation
FC: Fold Change
TMT: Tandem Mass-Tag
LF: Label-Free
LC-MS: Liquid Chromatography-Mass Spectrometry
SD: Standard Deviation
Dpi: Days Post Immunization
KO 0: *Peli1* Knock Out before EAE Immunization
KO 10: *Peli1* Knock Out 10 Days after EAE Immunization (disease onset)
KO 20: *Peli1* Knock Out 20 Days after EAE Immunization (disease peak)
WT 0: Wild-Type before EAE Immunization
WT 10: Wild-Type 10 Days after EAE Immunization (disease onset)
WT 20: Wild Type 20 Days after EAE Immunization (disease peak)
