# Supplementary material for: The Brain Proteome of the Ubiquitin Ligase Peli1 Knock-Out Mouse during Experimental Autoimmune Encephalomyelitis
Source: J Proteomics Bioinform. Author manuscript; Available in PMC 2016 Oct 12. (PMC5061044; doi:10.4172/jpb.1000408)
Supplement: Supp information [file NIHMS820356-supplement-Supp_information.pdf]

SUPPORTING INFORMATION

Pdf file: Graphical Abstract, Supplementary Figure 1. Clinical scores of *Peli1* KO and WT mice before and during EAE, Supplementary Methods.

Excel file 1: Supplementary Table 1. Analyses of the TMT labeling and LF proteomics data.

Excel file 2: Supplementary Table 2. Proteins regulated between *Peli1* KO 0 and KO 10 and/or WT 0 and WT 10.

Excel file 3: Supplementary Table 3. Proteins regulated between *Peli1* KO 10 and KO 20 and/or WT 10 and WT 20.

Excel file 4: Supplementary Table 4. Proteins regulated between *Peli1* KO 0 and KO 20 and/or WT 0 and WT 20.

Excel file 5: Supplementary Table 5. Standard deviation values and average abundances for each of the proteins used in figures
