# Supplementary material for: The Brain Proteome of the Ubiquitin Ligase Peli1 Knock-Out Mouse during Experimental Autoimmune Encephalomyelitis
Source: J Proteomics Bioinform. Author manuscript; Available in PMC 2016 Oct 12. (PMC5061044; doi:10.4172/jpb.1000408)
Supplement: supp fig [file NIHMS820356-supplement-supp_fig.pdf]

SUPPLEMENTARY FIGURES

Graphical abstract.

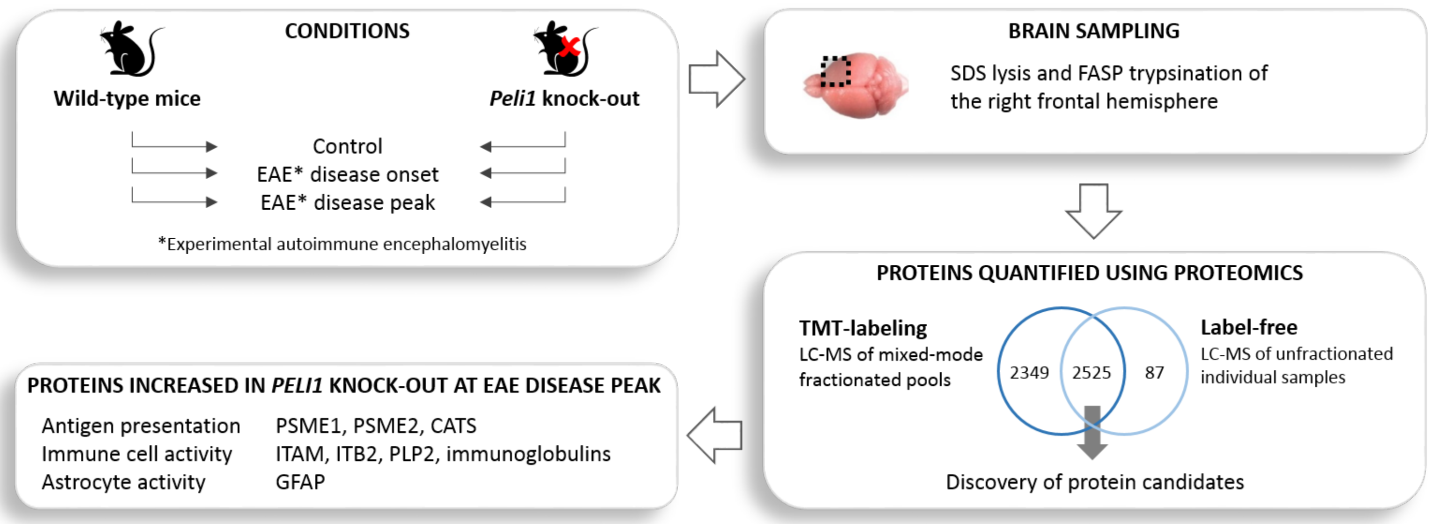

Supplementary Figure 1.

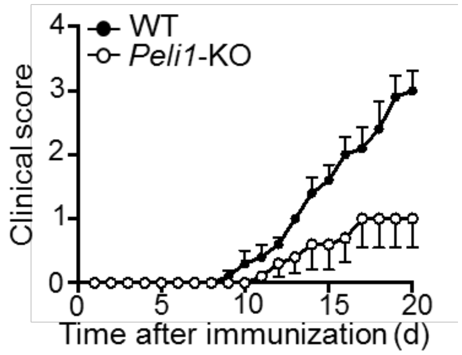

Average EAE clinical scores of the mice used in the study (n=38 at timepoint 0, n=28 at 10 days of EAE, n=14 after 20 days of EAE).
