# Supplementary material for: The Brain Proteome of the Ubiquitin Ligase Peli1 Knock-Out Mouse during Experimental Autoimmune Encephalomyelitis
Source: J Proteomics Bioinform. Author manuscript; Available in PMC 2016 Oct 12. (PMC5061044; doi:10.4172/jpb.1000408)
Supplement: supp methods [file NIHMS820356-supplement-supp_methods.pdf]

SUPPLEMENTARY METHODS

Biological samples

In normal condition, *Peli1* KO and WT mice had no weight difference, and age- and sex-matched mice were used for the EAE induction. Brains were harvested from mice euthanized before EAE immunization (0 days, n=5 for *Peli1* KO and n=5 for WT), and after 10 days (inflammation onset, n=7 for *Peli1* KO and n=7 for WT) and 20 days (disease peak, n=7 for *Peli1* KO and n=7 for WT) post immunization.

TMT labelling and sample grouping

The samples were pooled accordingly prior to digestion (**Table 1**). 50 µg of the 20 pooled samples were digested with the FASP method, desalted using OASIS plates and dissolved in 0.1 M TEAB. One TMT 10-plex was split in two and used to tag 20 samples, i.e 18 pools and two reference samples (9 pools and 1 reference each 10-plex experiment). The reference consisted of equal amounts of all the 38 samples. The 10 TMT 10-plex vials were added 41 µl anhydrous acetonitrile each, vortexed and incubated for 5 min at RT. Each vial was used to label two samples, one sample in each of the two 10-plex experiments. The 20 samples, added the respective TMT-reagent, were incubated for 1h at RT. The tubes were then added hydroxylamine to a final concentration of 0.3% (v/v) and incubated for 15 min at RT. The 10 samples tagged by one half of the TMT-10 plex were mixed in one tube and the 10 samples tagged by the other half were mixed in another tube, giving two 10-plex samples.

Table 1. TMT labeling of *Peli1* KO and WT brain sample pools.

***Peli1* KO**			**WT**			**Reference**
**0**	**10 dpi**	**20 dpi**	**0**	**10 dpi**	**20 dpi**	
2 samples TMT126 (KO 0 A)	2 samples TMT127C (KO 10 A)	2 samples TMT128C (KO 20 A)	2 samples TMT129C (WT 0 A)	2 samples TMT130N (WT 10 A)	2 samples TMT130C (WT 20 A)	38 samples TMT131
2 samples TMT127N (KO 0 B)	2 samples TMT128N (KO 10 B)	3 samples TMT129N (KO 20 B)	2 samples TMT128N (WT 0 B)	2 samples TMT128C (WT 10 B)	3 samples TMT129N (WT 20 B)	38 samples TMT131
1 sample TMT126 (KO 0 C)	3 samples TMT127N (KO 10 C)	2 samples TMT127C (KO 20 C)	1 samples TMT129C (WT 0 C)	3 samples TMT130N (WT 10 C)	2 samples TMT130C (WT 20 C)	

One TMT 10-plex kit was split into two subsets, experiment 1 (white background, 9 samples and 1 reference) and experiment 2 (grey background, 9 samples and 1 reference). The number of individual samples for the respective TMT-label is given in the table. The name of the sample used in data analyses is given in parenthesis. The two references consisted of an equal portion of all 38 samples and were identical for the two 10-plex subsets, enabling merging and comparison of the two 10-plex experiments in the data analysis.

Mixed-mode HPLC fractionation of TMT-labeled peptides

The two dried TMT tagged peptide samples were each resuspended in 300 µl Buffer A (20 mM ammonium formate in HPLC grade H_2O filtered through 0.45 µm pore size filter and adjusted to 3% ACN and to pH 6.5 with FA). A volume corresponding to 120 µg from each of the 10-plex samples were fractionated separately on a mixed-mode Promix MPcolumn (Promix MP-21.250.0530, 1x25mm 5µm 300Å, Scantec lab Sielc) using a 1260 Infinity Mixed mode LC-system (Agilent technologies). Buffer A was mixed with an increasing gradient of buffer B (2 mM Ammonium formate in HPLC grade H_2O, filtered as described for buffer A, adjusted to 80% ACN and adjusted to pH 3 with 50% FA) during a 70 min gradient, and the peptides were distributed across 60 fractions collected in a 96-well LoBind Eppendorf elution plate. The fractionation was carried out with a constant flow of 50 ml/min, with a gradient of 15% B from 0-10 min, then 15-60% B from 10-45 minutes, then 45-100% B from 45-55 min, and held stable at 100% until 60 min, then 100-15% from 60-65 min, and 15 % B from 65-70 min. Following fractionation, the samples were lyophilized and dissolved in 10 µl 5% FA and fraction 1-3 and 58-60 was pooled resulting in 56 samples each TMT 10-plex experiment.

LC-MS analysis of TMT-labeled samples

About 0.5 µg tryptic peptides dissolved in 5% aqueous FA were injected into an Ultimate 3000 RSLC system (Thermo Scientific) connected to an LTQ-Orbitrap Elite equipped with a nanospray Flex ion source (Thermo Scientific). Peptides were separated during a biphasic ACN gradient from two nanoflow UPLC pumps (flow rate of 280 nl/min) on the analytical column (Acclaim PepMap 100, 15 cm x 75 µm i.d. nanoViper column, packed with 2 µm C18 beads). Solvent A was 0.1% FA with 2% ACN and solvent B was 90% ACN. The mass spectrometer was operated in the data-dependent-acquisition mode to automatically switch between full scan MS and MS/MS. Instrument control was through Tune 2.7 and Xcalibur 2.2. Survey full scan MS spectra (from 300 to 2000 m/z) were acquired in the Orbitrap with resolution R = 120000 at 400 m/z (after accumulation to a target value of 1E6 in the linear ion trap with maximum allowed ion accumulation time of 300 ms). The 10 most intense eluting peptides above an ion threshold of 5000 counts and charge states 2 or higher, were sequentially isolated in the high-pressure linear ion trap to a target value of 5E5 at a maximum allowed accumulation time of 300 ms, and isolation width maintained at 2.5 Da. Fragmentation in the HCD (Higher-Energy Collision Dissociation) cell was performed with a normalized collision energy of 40%, and activation time of 0.1 ms. Fragments were detected in the orbitrap at a resolution of 30000 with first mass fixed at m/z 100. Fraction 1-11 were analyzed with a gradient of 5% B 0-5 min, then 5-12% B 5-60 min, 12-30% B from 60-90 min, 30-90% B from 90-100 min, 90% B from 100-105 min, 90-5% B from 108-120 min. Fractions 12-36 were analyzed with a gradient of 5% B from 0-5 min, 5-8% B from 5-5,5 min, 8-20% B from 5.5-60 min, 20-35% B from 60-90 min, 90-100% B from 90-100 min, 90% B from 100-105 min, 90-5% B from 105-108 min, 5% B from 108-120. Fractions 37-60 were analyzed with a gradient of 5% B 0-5 min, 5-8% B from 5-5,5 min, 8-40% B from 5,5-90 min, 40-90% B from 90-100 min, 90% B from 100-105 min, 90-5% B from 105-108 min, and 5% from 108-120 min.

Quantitation of TMT data in proteome discoverer

LC-MS data from the two TMT-10 plex experiments were analyzed in Proteome Discoverer 1.4 (Thermo Scientific) using Sequest and MS Amanda (version 1.4.4.2822) with the SwissProt *Mus musculus* database ver 27.03.14 (canonical sequence, no isoforms). Trypsin was set as enzyme, and maximum two missed cleavages were allowed. TMT tagging of N-teminal and lysines were set as a fixed modification, in addition to carbamidomethylation of cysteine. Oxidation of methionine was set as a variable modification. The fragment mass tolerance was set to 0.005 Da, and the identification deviance was set to 10 ppm for MS1 precursors. The PSM validation from both search engines was performed by Percolator, with a strict and relaxed target FDR of 0.01 and 0.05, respectively. The quantification method was TMT 10-plex with the integration tolerance of 20 ppm and the integration method was set to "most confident centroid". The mass analyser was FTMS, and the activation type was HCD. The minimum and maximum collision energies were 0 and 100, respectively.

Label-free sample preparation

All individual samples (n=38) used in the TMT experiment were analyzed by label-free proteomics. 20 µg of each individual sample, and 4 20 µl aliquots of a pool containing all the samples were digested by FASP and desalted by OASIS clean-up as described for the TMT experiment. Following lyophilization, the samples were dissolved in 1%FA, 2%ACN prior to LC-MS analysis.

Label-free LC-MS analysis

The reference samples were injected 4-7 times each for LC-MS analysis. Each run contained a survey full scan covering the 300 to 2000 m/z range to acquire all MS1 features within that range, and one of 12 different precursor mass range fragmentation windows of overlapping segments (10 m/z): 1) 300-460 m/z, 2) 450-510 m/z, 3) 500-560 m/z, 4) 550-610 m/z, 5) 600-660 m/z, 6) 650-710 m/z, 7) 700-760 m/z, 8) 750-810 m/z, 9) 800-860 m/z, 10) 850-910 m/z, 11) 900-1010 m/z and 12) 1000-2000 m/z. This was repeated twice, i.e a total of 24 runs. For the individual brain lysate samples the precursor fragmentation window was 300-2000 m/z. All individual samples were randomized prior to LC-MS/MS. The label free samples were analyzed using the same LC-MS system as for the TMT 10-plex analysis, with the following exceptions: The peptides were separated over a 195 min gradient. The percentage of solution B was held constant at 5% from 0 to 5 min, increased to 7% up to 6 min, increased to 32% up to 135 min, increased to 90% up to 150 minutes and held to 170 minutes. Solution B was decreased to 5% at 175 min and held at 5% until 195 min. Survey full scan MS spectra were acquired for 170 min in the Orbitrap with a resolution R = 240000 at 400 m/z (after accumulation to a target value of 1E6 in the linear ion trap with maximum allowed ion accumulation time of 300 ms). The 10 most intense eluting peptides above an ion threshold of 3000 counts, and charge states of plus 2 or higher, were sequentially isolated to a target value of 1E4 and fragmented in the high-pressure linear ion trap by low-energy CID (collision-induced-dissociation) with normalized collision energy of 35% and wideband-

activation enabled. The maximum allowed accumulation time for CID was 150 ms, the isolation width maintained at 2 Da, activation q = 0.25, and activation time of 10 ms. The resulting fragment ions were scanned out in the low-pressure ion trap at normal scan rate, and recorded with the secondary electron multipliers. One MS/MS spectrum of a precursor mass was allowed before dynamic exclusion for 18s. Lock-mass internal calibration was not enabled.

Label-free Quantification in Progenesis QI

The LC-MS/MS raw files were analyzed in Progenesis QI, ensuring an MS1 alignment score of >75% for all runs. Reruns of reference samples were included if the alignment of the MS1 features was high. Only unique peptides with charges from + 2 to +7 were used for quantitation. The results were exported from Progenesis QI as an mgf file. The mgf file was identified in Proteome Discoverer 1.4 as for the TMT 10-plex analysis, with the following exceptions: the database used was the Swissprot *Mus musculus* downloaded 15.10.2015 (canonical sequence, no isoforms) including the cRAP database contaminant sequences (ftp://ftp.thegpm.org/fasta/cRAP/). The fragment mass error was set to 0.7 Da. The modifications included were carbamidomethylation of cysteine as a fixed modification, and oxidation of methionine as variable. The identification results were exported from Proteome discoverer as a pep.xml file. Following import of identifications into Progenesis QI and assignment to the MS1 features, the protein quantification data was exported for further analysis in Microsoft Excel. The normalized protein intensities for each of the individual samples were divided by the median intensity of the reference samples, and these ratios were $\log_2$ transformed prior to statistical analyses and fold change calculations.
